# MLCA—A Machine Learning Framework for INS Coarse Alignment

**DOI:** 10.3390/s20236959

**Published:** 2020-12-05

**Authors:** Idan Zak, Reuven Katz, Itzik Klein

**Affiliations:** 1 Autonomous Systems Program, Technion–Israel Institute of Technology, Haifa 3200003, Israel; 2 Faculty of Mechanical Engineering, Technion–Israel Institute of Technology, Haifa 3200003, Israel; reuvenk@technion.ac.il; 3 Department of Marine Technologies, University of Haifa, Haifa 3498838, Israel; kitzik@univ.haifa.ac.il

**Keywords:** inertial navigation system, coarse alignment, machine learning

## Abstract

Inertial navigation systems provides the platform’s position, velocity, and attitude during its operation. As a dead-reckoning system, it requires initial conditions to calculate the navigation solution. While initial position and velocity vectors are provided by external means, the initial attitude can be determined using the system’s inertial sensors in a process known as coarse alignment. When considering low-cost inertial sensors, only the initial roll and pitch angles can be determined using the accelerometers measurements. The accuracy, as well as time required for the for the coarse alignment process are critical for the navigation solution accuracy, particularly for pure-inertial scenarios, because of the navigation solution drift. In this paper, a machine learning framework for the stationary coarse alignment stage is proposed. To that end, classical machine learning approaches are used in a two-stage approach to regress the roll and pitch angles. Alignment results obtained both in simulations and field experiments, using a smartphone, shows the benefits of using the proposed approach instead of the commonly used analytical coarse alignment procedure.

## 1. Introduction

An inertial navigation system (INS) is a dead reckoning (DR) navigation system that integrates the outputs of the inertial sensors to calculate the current position, velocity, and attitude of a platform without any external aids [1]. The INS is a self-contained navigation system without the need for external signals or communication, comprising an inertial measurement unit (IMU) and a navigation processor. Most autonomous platforms employ INS as their main navigation sensor, mainly because it is a low-cost standalone system that can provide the required navigation data for the autonomous operation: position, velocity, and orientation. The IMU inertial sensors typically consist of an accelerometer triad (three mutually orthogonal accelerometers) that measure the specific force vector and a gyroscope triad aligned with the accelerometers that measure the angular rate vector [2]. The navigation processor uses the raw measurements of the inertial sensors (after some gravity corrections) to produce the navigation solution of the position, velocity, and orientation of the vehicle. However, due to errors in the measurements, the solution of the INS diverges with time. The rate of divergence depends on INS quality. To circumvent the navigation solution drift INS are commonly fused with external sensors [3,4].

In a strapdown inertial navigation system (SINS) implementation, the accelerometers and gyros are mounted rigidly onto the platform, which requires less power consumption, and with much smaller weight and dimensions than other gimbaled implementations. Current INS systems are commonly based on micro-electro-mechanical-system (MEMS) technology that applies small and low-priced sensors but with relatively high errors that can dramatically affect the overall navigation solution performance [5,6,7]. The MEMS INS is the most frequently used SINS technology.

As a DR system, it must have the initial navigation state conditions before initiating the navigation solution. The initialization of the position and velocity vectors is made using external sensors or information (such as from global navigation satellite systems). The initial attitude, however, can be determined using inertial sensors [7,8,9]. This process starts with a vital step of coarse alignment (CA), whose purpose is to calculate the initial attitude angles: roll and pitch from the accelerometer’s readings and yaw from the gyroscope’s readings. For low-cost inertial sensors only, the roll and pitch can be determined from the accelerometers in stationary conditions [2,10]. When completing the CA procedure, commonly fine alignment (FA) is immediately applied to improve the CA accuracy. To that end, FA uses external sensors or information, such as zero velocity updates, in a fusion process to improve the attitude accuracy [2,8]. Recently, an analytic evaluation of the steady-state properties of the FA process was derived [11].

Recently, several studies have addressed the alignment process in order to reduce misalignment errors and increase performance. Ref. [12] shows the treatment of the noise in the outputs of the IMU, and [13] propose improved signal denoising methods. Some other novel initial alignment algorithms for autonomous underwater vehicles (AUVs) were proposed [14,15,16] based on an improved fine alignment that requires lower CA accuracy. Instead of the traditional navigation system model and extended kalman filter (KF), a comprehensive study of nonlinear filter methods, like), unscented Kalman filter, and particle filter, are addressed [17]. Other papers introduce methods based on additional sensors data doppler velocity [18] for accurate fine alignment. 

The promising field of machine learning (ML) is penetrating into the worlds of navigation—such as indoor navigation in [19,20,21], localization based on inertial sensors based on dynamic detection of situations of interest in [22]. The use of a combined support vector machine was proposed in [23], which applied to the initial alignment of INS and did show better performance than the traditional Kalman filtering approach. A deep-learning model for inferring the momentary speed in inertial navigation systems was proposed in [24]. A Convolutional Neural Network (CNN) methodology is described in [25] to remove error sources in the inertial sensor signals. Another recent work [26] proposes a method with which to navigate by using data from low-grade INS sensors (accelerometers and gyroscopes) on a moving vehicle by employing ML techniques instead of the traditional method of using a KF. However, not much research has been done on the inertial navigation alignment problem. 

In this paper, we aim to fill this gap and propose an ML-based CA methodology for low-cost sensors. We aim to demonstrate the ability to predict the initial roll and pitch angles, using computational learning algorithms, given the inertial sensors readings and a priori database containing the system behavior from other previously recorded alignment scenarios. We present a new methodology of MLCA, a machine-learning-based CA, and the MLCA Pyramidal Methodology approach to cope with computing hardware limitations. In the prediction solution, we show improvement in the accuracy and time required for the alignment process, making it possible to use learning algorithms to replace the classical CA with a computational learning alignment method. A very preliminary work of this research was published in [27], while this paper elaborates it with broader research scenarios, methodology, and real experimental results. The development of this new ability to perform the process of aligning the INS quickly and accurately by smart integration of ML algorithms can constitute a breakthrough in many applications and platforms, specifically those operating in pure inertial navigation conditions.

## 2. Problem Formulation

### 2.1. INS Navigation Equations

While navigating, the INS determines the updated position, velocity, and orientation by solving the navigation equations of motion. These equations provide the position, velocity, and orientation rate of change in the navigation frame. 

The position vector is expressed in the navigation frame using latitude/longitude/height (LLH) formulation [1]:

(1) $$r^{n}={\left[\begin{array}{ccc} \phi_{L} & \lambda & h \end{array}\right]}^{T}$$

The velocity vector, as expressed in the navigation frame using the north-east-down coordinates, is:

(2) $$v_{}^{n}={\left[\begin{array}{ccc} v_{N} & v_{E} & v_{D} \end{array}\right]}^{T}$$

The rate of change of the position and velocity vectors in the navigation frame is given by:

(3) $${\dot{v}}^{n}=T_{b}^{n}f_{ib}^{b}+g_{}^{n}-\left(2\omega_{ie}^{n}+\omega_{en}^{n}\right)v^{n}$$

where $g_{}^{n}$ is the gravity vector expressed in the navigation frame, $\omega_{ie}^{n}$ and $\omega_{en}^{n}$ are the angular velocities vectors between the earth-centered-earth-fixed (ECEF) frame and inertial frame and between the navigation frame and the ECEF frame, respectively, both expressed in the navigation frame and $f^{b}$ is the specific force vector as measured by accelerometers and expressed in the body frame given by:

(4) $$f_{ib}^{b}=\left[\begin{array}{ccc} f_{x} & f_{y} & f_{z} \end{array}\right]$$

The body to navigation transformation matrix, $T_{b}^{n}$, rate of change is given by:

(5) $${\dot{T}}_{b}^{n}=T_{b}^{n}\omega_{ib}^{b}-\left(\omega_{ie}^{n}+\omega_{en}^{n}\right)T_{b}^{n}$$

where $\omega_{ib}^{b}$ is the angular velocity vector as measured by the gyros. The transformation matrix from the navigation frame to the body frame is as follows [1]:

(6) $$T_{n}^{b}=\left[\begin{array}{ccc} c(\theta )c(\psi ) & c(\theta )s(\psi ) & -s(\theta )\\ s(\phi )s(\theta )c(\psi )-c(\phi )s(\psi ) & s(\phi )s(\theta )s(\psi )+c(\phi )c(\psi ) & c(\theta )s(\phi )\\ c(\phi )s(\theta )c(\psi )+s(\phi )s(\psi ) & c(\phi )s(\theta )s(\psi )-s(\phi )c(\psi ) & c(\theta )c(\phi ) \end{array}\right]$$

For low-cost sensors and in stationary conditions, $\omega_{ie}^{n}$ and $\omega_{en}^{n}$ are neglected in (3) and (5).

### 2.2. Strapdown INS Alignment 

While the initial position and velocity are determined by external sensors, an initial alignment process is required to obtain the initial orientation. There, the IMU measurements are used to determine the transformation matrix from the b-frame to the n-frame, i.e., the initial roll, pitch, and yaw angles [1,2]. The initial alignment typically consists of two stages: CA and a FA [1,28,29]. The CA is a process whose objective is to provide a good condition for the FA stage. There, the misalignment angles are approximately estimated, without any external aids. In the FA stage, the initial attitude is used to initiate a nonlinear filter, and using external aids, it aims to improve the orientation accuracy. For commonly used low-cost inertial sensors, only the initial roll and pitch angles can be determined using the accelerometers during a stationary CA stage [2,10]. 

**Figure 1:**
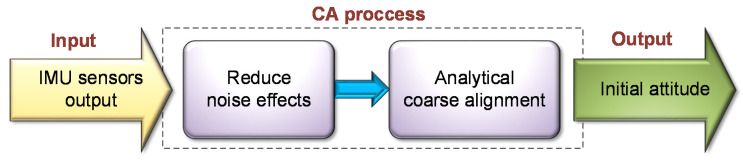
Traditional INS CA process.

The basic concept of the CA is to determine the initial orientation by using the known value of the gravity vector (and for high-end sensors also the known value of the earth turn rate). The traditional CA process of the INS is illustrated in Figure 1. In strapdown systems, the CA is conducted analytically, and it consists of two main steps: the first step is some pre-processing of the input sensors readings whose purpose is to reduce the noise level. Secondly, the initial values of the roll and pitch attitudes are calculated using an analytical transformation of the values to the current attitude of the system [1]. This process is described below.

For stationary alignment, the acceleration in the navigation frame equals zero, that is, ${\dot{v}}^{n}=0$. Thus, using (3) and (6) it is obtained [2]:

(7) $$f_{ib}^{b}=\left[\begin{array}{c} f_{x}\\ f_{y}\\ f_{z} \end{array}\right]=T_{n}^{b}\left[\begin{array}{c} 0\\ 0\\ g \end{array}\right]=\left[\begin{array}{c} s(\theta )\\ -c(\theta )s(\phi )\\ -c(\theta )c(\phi ) \end{array}\right]g$$

where *θ* is the pitch angle and *ϕ* is the roll angle. 

The pitch and roll angles may be determined analytically from (7):

(8) $$\theta =\arctan\left(\frac{-f_{ib,x}^{b}}{\sqrt{f_{ib,y}^{b}{}^{2}+f_{ib,z}^{b}{}^{2}}}\right)$$

(9) $$\phi =\arctan2(-f_{ib,y}^{b},-f_{ib,z}^{b}{}_{z})$$

In practice, the values of the force vector $f_{ib}^{b}$ in (8) and (9) are calculated by taking the mean values of the accelerometer measurements for a given time period. The CA process precision and time directly affect the overall navigation system performance, in particular for situations of pure inertial navigation. Incorrect attitude initial conditions will result in erroneous position and velocity while an improved alignment precision reduces the navigation errors, particularly for situations of pure inertial navigation.

## 3. Machine Learning Based CA Methodology

### 3.1. Approach

**Figure 2:**
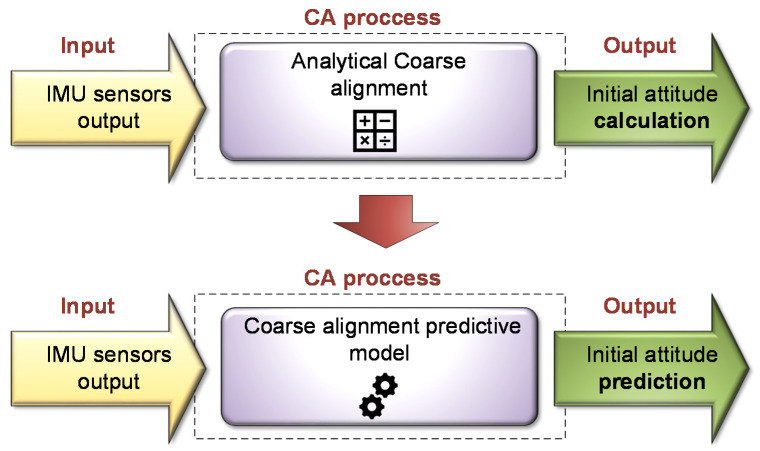
Proposed INS CA process using ML.

The basic idea of the proposed approach is to replace traditional CA process with a new one, based on a pre-learned CA predictive ML model, namely MLCA. The proposed MLCA approach is illustrated in Figure 2. The CA task is treated as a supervised, regression machine learning problem. Given the accelerometer readings, the trained ML classifier outputs the roll and pitch angles.

For the training dataset, the accelerometer measurements were obtained from simulations. There, in stationary conditions, the measurements were modeled by nominal values and an additive velocity random walk error. In addition to the noisy accelerometer measurements, the corresponding target labels, the true values of the roll and pitch angles, were constructed using the nominal accelerometer readings that were substituted into (8) and (9). 

**Figure 3:**
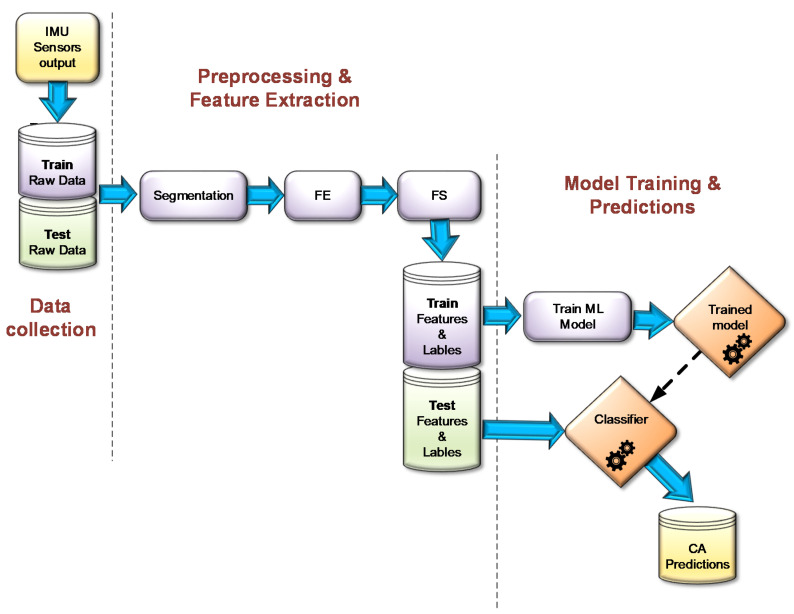
MLCA model training and testing process.

The set of accelerometer measurements are addressed as time-series data [30,31]. Moreover, the IMU raw data is, actually, a multivariate time-Series data since it includes more than one feature, which varies along the timeline. Given the raw data, it is critical to identify some strong features for the success of the trained phase in the prediction task. In order to calculate features from the raw data, pre-processing work was done on the given time series, in which a sliding window method was used to split the whole data set into window segments. Each window has a fixed width of samples, with overlaps between consecutive windows. For each window, a suitable set of features (Section 3.3) was calculated in the feature extracting stage (FE). Then, a feature selection (FS) method was applied in order to discard the irrelevant or redundant features, thus selecting only the important subset of features to be applied in the final stage of the modeling process [32]. A greedy optimization strategy was applied in order to find the best performing subset of features by running the recursive feature elimination (RFE) wrapper FS [33,34] method. This training process is presented in Figure 3. This approach is applicable for any set of required roll and pitch angles and with any resolution. 

The selection of the window width has a high significance, as it determines the amount of accelerometer raw data that is used to calculate our features. The actual total amount of accelerometer raw data that needed to accumulate before the roll and pitch can be predicted is determined by the prediction time. Note that the size of the window can be different for given prediction time values, and its maximum width is bounded by the prediction time value. Using a smaller window width allows short prediction for a faster alignment process, which requires less time to converge but can influence the accuracy. To determine the optimal window duration value and number of sample overlaps, a range of widths for each of the ML methods was tested and compared the results with the traditional alignment method, as presented in Section 4. 

Six regression-based ML algorithms are employed in this research for the purpose of MLCA. random forest (RF) [35], extremely randomized trees (ExtraTrees) [36], a more recently developed ensemble methods of extreme gradient boosting (XGBoost) [37], Light gradient boosting machine (LightGBM) [38], and category boost (CatBoost) [39]. The ensemble method is a major learning paradigm that combines the hypotheses predictions from a collection or ensemble of machine learning algorithms to get a more accurate prediction than each of the individual models [40]. These ML techniques used to predict the roll and pitch CA initial angles and analyze the relative performance of those algorithms versus the classical CA. Random Search and Grid Search methods were both used to optimize each of the ML model’s hyper-parameters to our data sets. Random Search tests the model performance for random combinations for the hyper-parameters from a given values sets. This can help us focus on the relevant values ranges. Then a Grid Search can be used, which tests all the combinations for a given value sets for each of the hyperparameters in the ranges we focused on. This process results with the best hyper-parameters that should be used when training each model.

### 3.2. MLCA Pyramidal Methodology

Although the proposed approach can be used for any range of angles, in practice, the training stage may be limited by the hardware employed. Training machine learning models on data sets with a large number of samples is a challenging problem due to the memory and CPU limits of the computing hardware. In our case, the scale of the data set is directly influenced by the angles range and angle resolution. Therefore, working with an angle wide range and with high-resolution was not an option. To circumvent this problem, a pyramidal methodology for the training stage is employed. There, a two-level CA is applied. In the first phase, an attitude prediction of a wide range and low-resolution model allows to focus on a specific area of angles on which, in the next phase, the matching narrow range and high-resolution model is applied on the IMU raw data for high accuracy attitude prediction. That is, the narrow-range model does not run on the output of the wide range model, but rather the relevant narrow range model is selected by this prediction output. 

**Figure 4:**
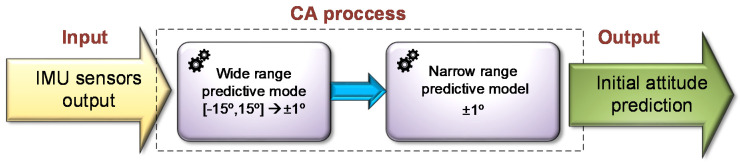
Proposed MLCA pyramidal process- wide range followed by narrow range regression.

This pyramidal concept is illustrated in Figure 4. Herein, we defined the wide range of initial roll and pitch angles to be in the range of 0 ± 15 deg with resolution of 1 deg and 0.5 deg while the narrow range is in the range of 0 ± 1 deg with resolution of 0.05 deg and 0.01 deg. Yet, of course, the proposed approach can be applied on any set of angles and resolutions pending the hardware the training is applied. Once the classifier is trained, it can be used with low computational requirements.

Notice that, for evaluating this proposed approach, the performance tests of models in narrow-range are independent from the wide-range tests outputs, they are trained and tested on higher resolution data in narrow range. The wide-range models performance tests should show the ability to focus on the relevant narrow range model.

### 3.3. Features Description

A total of 18 features types were used on the accelerometer’s readings. Among them, 14 statistical features, two time domain features, one frequency-domain feature, and one cross-correlation feature. Definition of the extracted features are presented in Table 1, Table 2, Table 3 and Table 4, where $x_{i}$ is the accelerometer reading at index *i* in the window, and *N* is the total number of samples used. The statistical(including histogram of four bins), time domain, and frequency-domain features (total of 20) were computed for each accelerometer axis within each window (60 features), and 10 of them were also computed on the magnitude of the specific force vector. Additionally, three accelerometer axis correlation features results with a total of 73 features used in the analysis data sets.

### 3.4. Evaluation Metric 

To compare between the traditional CA and the MLCA, a common metric that is used to measure accuracy for continuous variables was employed: the mean absolute error (MAE). The MAE metric is a natural measure of average error and (unlike root mean square error, for instance) is unambiguous [41]. This metric will express the average model prediction error in units of the predicted angles (degrees/mrad). The MAE measures the average magnitude of all predictions errors:

(10) $$MAE=\frac{1}{n}\sum_{i=1}^{n}\left|y_{i}-{\hat{y}}_{i}\right|$$

where $y_{i}$ and ${\hat{y}}_{i}$ are the true reference value and predicted one for measurement i, and n is the number of measurements in the data set. In the case of CA, the total number of measurements to be used is defined by the required time for the prediction. For example, consider an accelerometer sampling at 100 Hz for the required prediction time of one second, will result in 100 measurements for the CA calculation. For performance analysis, the CA predictions error is calculated for all the sequential windows of 100 accelerometer measurements over the time series raw data, then the MAE is calculated over all those CA predictions. However, For the MLCA, the window size can be smaller than the prediction time. For example, if each time series is divided into windows of 75 measurements with overlaps of 74 between them, for each time sequence of 1 s prediction time, there are 25 windows. In this case, the MLCA prediction for the required prediction time of one second is calculated by the mean prediction value over those 25 windows, and then the MAE is calculated over all MLCA predictions for the entire test time series.

## 4. Results and Discussion

The proposed ML-based alignment methodology was evaluated using simulations and field experiments using smartphones. The following sections present the main simulative and experimental results, including classical CA and MLCA. The MLCA results presented in the tables are the ones obtained after feature selection, hyper-parameter tuning, and a window size optimization for each method.

### 4.1. Simulations 

A simulative environment was developed to emulate the accelerometers sensors readings of a low accuracy with a velocity random walk (VRW) error model:

(11) $${\tilde{f}}_{imu}=f_{true}+w_{a}$$

where $f_{true}$ is the nominal value of the specific force, and $w_{a}$ is the inertial sensors random noise defined as a zero-mean white Gaussian noise. To that end, a velocity random walk value of 0.05 $m/s/\sqrt{h}$ was used.

In stationary conditions, the values of the deterministic parts of the bias, scale-actor and misalignment error terms can be mostly removed. Therefore, those were not addressed in the simulation part. Later, in the experiments, all error-terms are accounted for. Using this simulation, noisy accelerometer readings were produced, each scenario for 30 s with a sampling rate of 100 Hz, labeled with the nominal reference of the roll and pitch angles. In total, 138.9 million samples (from the three accelerometers) were created, of which 138.3 million for the train datasets and 600 K for the test dataset.

Four train sets in two representative angles ranges, differing by their resolution, were chosen for the analysis: First, within a narrow range of angles −1° ≤ roll, pitch ≤ 1° with relatively high resolutions of 0.01 and 0.05 degrees, that contain 120 million and 4.8 million samples respectively, and then a wider range of −15° < roll, pitch < 15° in lower resolutions of 0.5 and 1 degrees that contain 10.8 million and 2.7 million samples respectively. For example, the narrow range train set of −1° ≤ roll, pitch ≤ 1° with a resolution of 0.01, contains 40 K combinations of 200 roll and 200 pitch angles. Each simulation was recorded for 30 s at 100 Hz, which means that each data set contains a total of 120 million measurements of the three-axis accelerometers. The test sets were comprised of new additional 100 simulations recordings, each for 30 s at 100 Hz, in the narrow and wide ranges, respectively.

The motivation for this strategy was to cope with the huge amount of information collected from the simulative accelerometers operating at 100 Hz, using a standard CPU computer and still evaluate the ability of MLCA both in a wide-angle range and also narrow range.

For narrow range tests, the ML models were first trained, twice, on the entire simulative train sets in the two resolutions of 0.05° and 0.01° for roll and pitch angles in the range of 0 ± 1°. The test set was comprised of new additional 100 simulations recordings, each with a different random orientation in the range of 0 ± 1° for the roll and pitch angles values. Given the train and test sets, a total of 73 features (as presented in Section 3.3) were calculated. Then, 6 ML approaches and the classical CA approach were applied on the data. 

Table 5 shows prediction results for the random orientations test set, as obtained from the ML models trained in the resolution of 0.01°, for a required for prediction duration of 3 s (300 accelerometer measurements). The traditional CA error results for the roll and pitch angles are 0.336 mrad and 0.323 mrad, respectively, while MLCA achieved better results in all methods. The maximum roll improvement was to 42.75% with the XGBoost method and 42.42% improvement for pitch angle when using the LightGBM method.

The results show a clear advantage of ML models predictions over the traditional CA method, where all models achieved improvement over the classical method results around 40% and more for both the roll and the pitch. 

**Figure 5:**
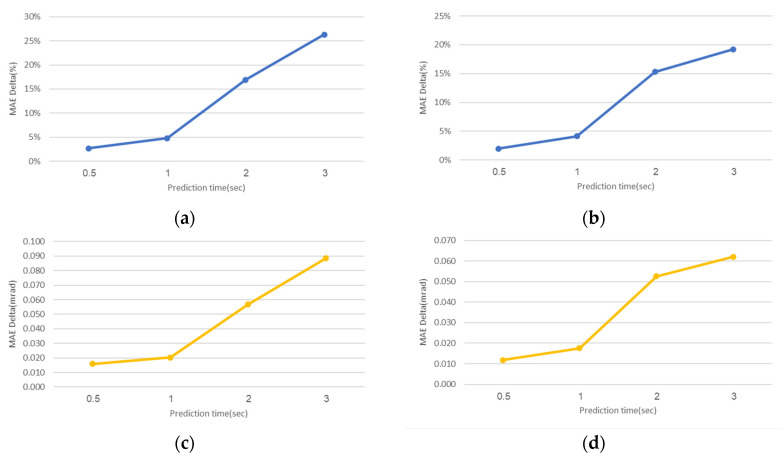
Narrow range delta error values using XGBoost CA vs. classical CA in various prediction times. (**a**) Roll error delta (%); (**b**) Pitch error delta (%); (**c**) Roll error delta (mrad); (**d**) Pitch error delta (mrad).

The required CA prediction time has a strong influence on the performance. In general, as the required time increases, the results improve both in the classical and ML approaches. However, as the CA time increases, the ML performance is improved compared to the classical CA method. Figure 5 shows the percentage of the improvement achieved by the XGBoost model, trained in the lower resolution of 0.05° in the range of 0 ± 1° for the roll and pitch angles, relative to traditional CA, for several prediction time durations. The model increased its MAE improvement from 3% and 2% for 0.5 s prediction time, for the roll and pitch angles, until it reached 26% in roll and 19% in pitch when the prediction time was 3 s. 

**Figure 6:**
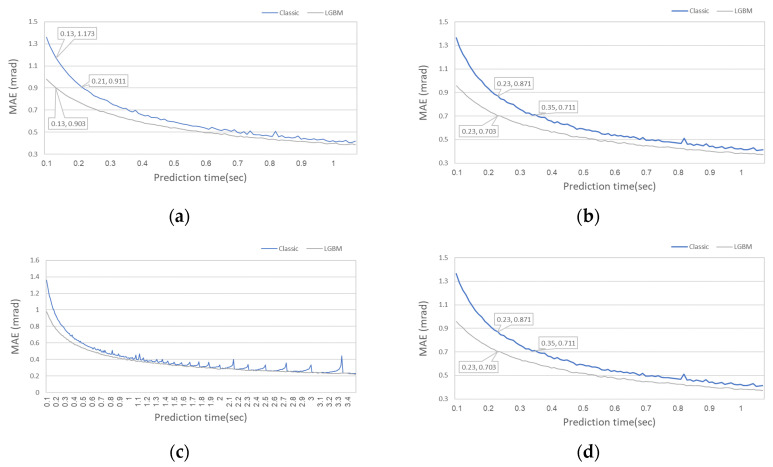
LightGBM compared to classical CA prediction errors vs. prediction time. (**a**) Roll error for prediction time in the range of 0.1–1 s; (**b**) Pitch error for prediction time in the range of 0.1–1 s; (**c**) Roll error for prediction time in the range of 0.1–3.5 s; (**d**) Pitch error for prediction time in the range of 0.1–3.5 s.

To better show the improvement that can be achieved using the ML CA, the LightGBM model performance is further evaluated. Figure 6 shows the roll and pitch prediction errors for the LightGBM model trained in the resolution of 0.01° in the range of 0 ± 1° for the time required for prediction in the range of 0.1–3.5 s, which corresponds to 10–350 accelerometer measurements in each axis.

The results shows two benefits of MLCA: (1) the ability to obtain lower errors at the same prediction time and (2) achieving a required CA error level in a shorter prediction time. For example, the traditional CA error result for the roll angle in Figure 6 is 1.173 mrad after 13 accelerometer measurements, while the MLCA using LightGBM obtained better results with only 0.9 mrad MAE, which is a 23% improvement. Moreover, the LightGBM MAE converged to 0.7 mrad after 23 accelerometer measurements while CA needs 35 measurements to achieve such accuracy, which is another 12 ms for having similar prediction accuracy. In some applications, it is a meaningful time duration. The LightGBM model also shows predictive stability across all prediction time values versus the classical method, which is heavily influenced by local errors in some of the higher prediction time values, and its MAE increases sharply.

After validating the ability to have a better prediction for the CA than the classical method in the narrow range, prediction of a wide range and low-resolution ML models is now addressed. ML models were trained on simulative train sets with roll and pitch values in two resolutions of 0.5° and 1° in a wide range of 0 ± 15°. The test stage was made with an additional 100 simulations set of recordings, each with a different random orientation in the range of 0 ± 15° for the roll and pitch angles values. Table 6 shows prediction results obtained from the ML models trained in the resolution of 0.5° and 1°, for the time required for the prediction of 3 s, which corresponds to 300 accelerometer measurements in each axis.

**Figure 7:**
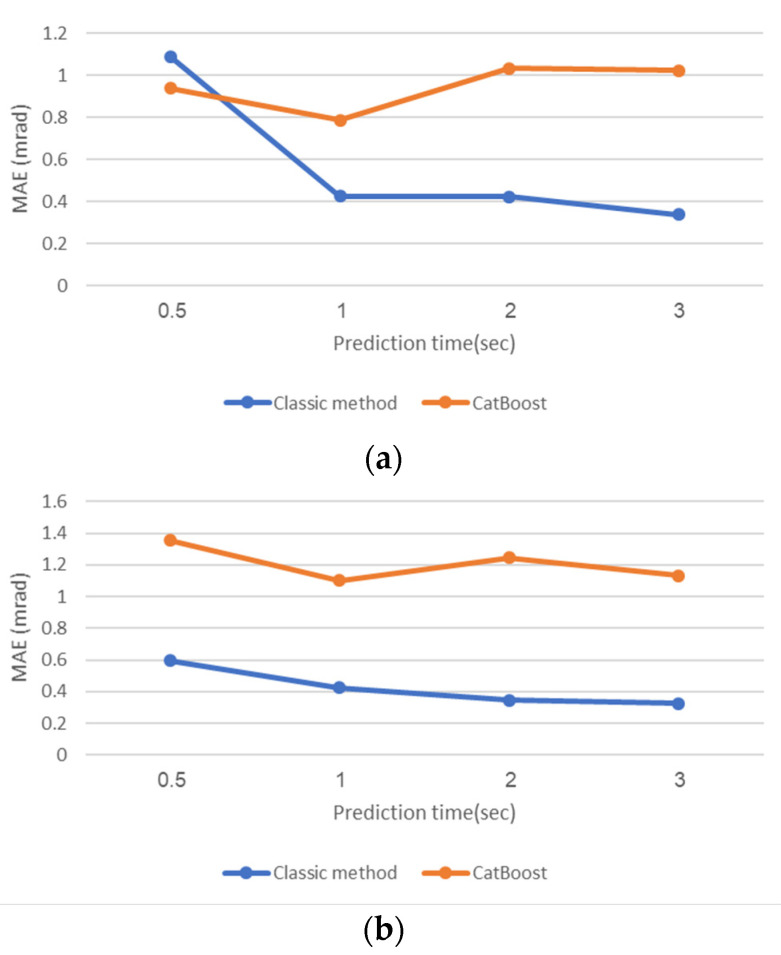
Wide range error values using CatBoost CA vs. classical CA. (**a**) Roll error; (**b**) Pitch error.

As seen in the narrow range, also in the wide range—increasing the train set angles’ resolution improves the results clearly and significantly. However, we are limited in the ability to raise the resolution due to the limitations of memory and CPU. However, still, the results show that there is a reasonable ability to predict the angles using the ML model in the wide range in all the methods tested, which enable us to focus the search in the relevant narrow range model as presented before. Referring to Table 6, for ML models trained in the resolution of 0.5°, the worse results for a 3 s prediction time are 2.03 mrad (±1.56) for the roll and 2.26 mrad (±1.1) for the pitch, which are 0.12° (±0.09) for the roll and 0.13° (±0.06), when XGBoost method was used. Those results can still easily guide us to the relevant narrow range model. Among the ML methods we tested in the wide range, there is generally an advantage to the CatBoost, LightGBM, and ExtraTrees methods, where CatBoost usually provides the best and most stable prediction. We can also see the effect in this wide range of the time required for prediction on both the classical method and the ML so that, in general, a long time allows for better results. Figure 7 shows this in a more detailed comparison of the classical method results versus the CatBoost method trained in the resolution of 0.5°.

To summarize, classical CA keeps the same level of performance both in a narrow and wide range. For MLCA, a wide range is used to direct and focus on the narrow range CA to yield the roll and pitch estimation. It was shown that the overall time required in MLCA to obtain the same accuracy is much lower than the traditional CA, and its overall accuracy is better.

### 4.2. Experiments

**Figure 8:**
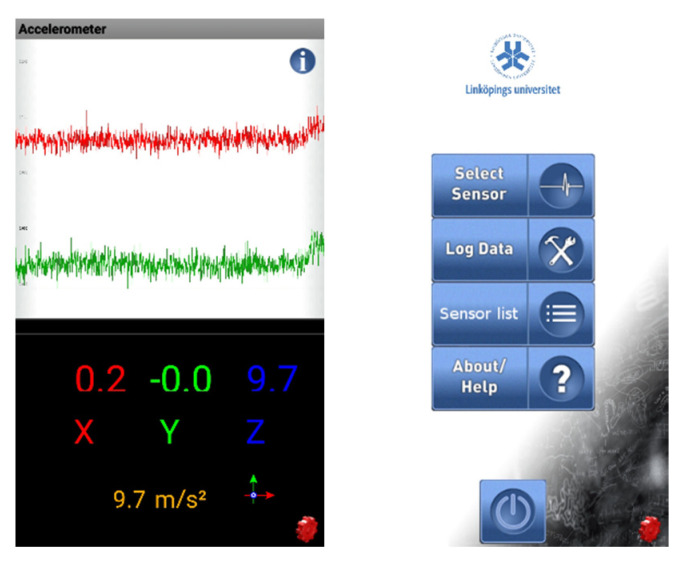
Snapshot of the sensor fusion app screens.

Field experiments were conducted using smartphone-based inertial sensors under real environment operation conditions. The MLCA predictive models have been tested in a set of stationary INS alignment scenarios. The sensor’s raw data was recorded using the ‘Sensor Fusion’ android application, which was developed at Linköping University (LiU) in Sweden [42]. The application screenshots are presented in Figure 8. The set of real inertial sensors measurements having their errors and random noise, which have been recorded, was then used as input for the performance comparison test between the traditional CA to the MLCA instead of the simulated data. The sensor fusion Android application was installed on the Samsung Galaxy phone and was configured to record the specific force vector and smaerphone orientation.

To calculate the attitude ground truth (GT) recordings of three minutes while in stationary conditions were made. There, the attitude solution provided by the application was employed. The attitude is calculated in an Attitude and Heading Reference System (AHRS) framework using the well-known Madgwick algorithm [43] which is based on both gyroscopes and accelerometer readings. This algorithm provided the attitude estimation for a time duration of three minutes, over each of the recorded raw time series, while the GT was taken as the average attitude value. By averaging, the influence of noise on the solution is reduced. Assuming zero-mean white Gaussian noise, as more samples are used the better is the noise reduction. Prior sensor calibration was not conducted; thus, misalignment errors were not removed. In the following experiments, we compared CA and MLCA for a time duration of one second, therefore the noise reduction has there less influence compared to the GT. Both traditional CA and the proposed MLCA were compared to the same GT. 

**Figure 9:**
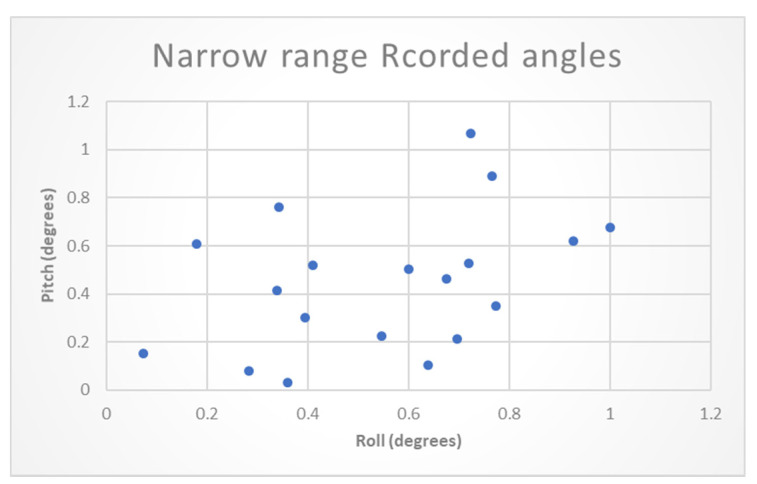
Experiment narrow range recorded angles distribution.

The MLCA performance in the field experiment was first evaluated in a narrow range of 0–1° for the roll and pitch angles. The ML models were first trained and tested on a dataset of raw data in a narrow range that contains 3-min random recordings of varying angles in the narrow range of 0–1°. Figure 9 presents the distribution of the recorded orientations. 70% of each of the recordings in the dataset was used as a training set, and the rest was used as the test set. 

Table 7 shows the experimental results for the low accuracy accelerometers in a narrow range scenario that were produced for one second prediction time, which corresponds to approximately 100 accelerometer measurements. The CA error results for the roll and pitch angles are 0.233 mrad and 0.255 mrad, respectively, while MLCA achieved significantly better results in all presented methods with up to 85.89% and 78.03% relative improvement for roll and pitch angles with the LightGBM method.

That is, working on the real experimental data sets, MLCA showed remarkable results with LightGBM been able to predict the roll and pitch angles better than the classical method on a known set of angles sets with up to 86% and 78% relative MAE improvement, while CatBoost did also well with up to 67% and 76% relative improvement for a one-second prediction time. 

Similar experimental results were also achieved with the same ML models in the narrow range trained on the full dataset of recordings in the range of 0–1° while the roll and pitch angles were tested against a newly recorded data set of a different orientation than the ones in the train set.

Table 8 presents the prediction results on a new set of recordings in randomly chosen orientation of 0.93° for the roll and 0.52° for the pitch angle produced for the time required for prediction of one-second. The CA error results for the roll and pitch angles are 0.177 mrad and 0.153 mrad, respectively, while MLCA achieved significantly better results in most presented methods with up to 70.94% relative improvement for the roll using LightGBM and 45.07% for the pitch angle when the RF method used. 

The results show that even when tested on new angles, there is a clear improvement in favor of the MLCA methods over the classical method. LightGBM and RF stood out with the best results and showed an accuracy improvement for new angles by 71% and 70% for the roll and by 45% and 37% for pitch, respectively, versus the classical CA. 

**Figure 10:**
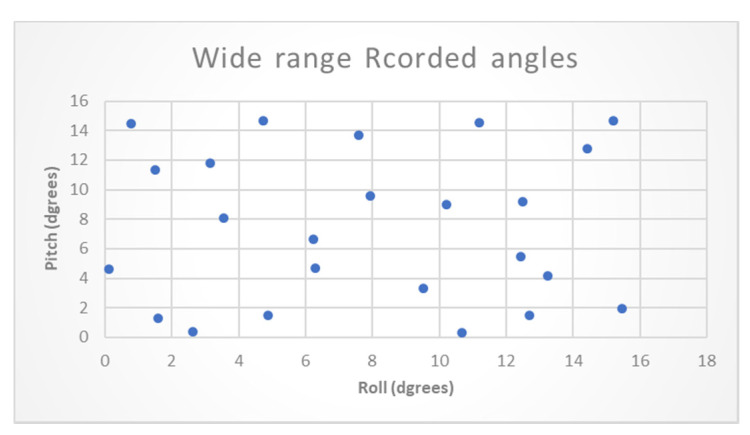
Experiment wide range recorded angles distribution.

Next, CA prediction in a wide range low-resolution dataset was evaluated. This is a necessary step in order to validate the possibility to later focus on a specific area of narrow range angles. At this stage, 3-min recordings were collected at varying angles in the wide range of 0–15°. Figure 10 presents the distribution of data set recordings in the wide range.

The ML models were initially trained and tested on this data set, where 70% of each of the recording in the dataset were used in the training set and the rest in the test set. Then, the same ML models in the wide range that were trained on the full dataset of recordings in the range of 0–15° for the roll and pitch angles were tested against a newly recorded data set of a different orientation than in the train set. Table 9 presents a comparison of the prediction results of ML models on a new random orientation recording of 10.88° for the roll and 3.01° for the pitch angle that was not present in the train set. The results were produced for the time required for the prediction of one-second. The comparison is between classical CA and MLCA, including the precision in terms of MAE results. 

Similar to the simulation results, the ML models trained in a wide range didn’t achieve better results than the classical method. But again, the achieved values, including the STD values, can easily allow us to focus on a narrow range and run the relevant model that has been trained for that range, with better resolution, and get overall better results (in shorter times)compared to the classical method. For example, from the results in Table 9 for the tested ML methods, the worse errors achieved when using CatBoost with MAE of 9.41 mrad (0.54° ± 0.26) for the roll and 5.202 mrad (0.3° ± 0.05) for the pitch. Furthermore, all other methods obtained much higher accuracy. For example, ExtraTrees achieved an accuracy of 2.076 (±0.22) mrad and 1.092 (±0.52) mrad for the pitch and roll angles, respectively, which are 0.12° (±0.01) for the roll and 0.06° (±0.03), which can easily guide us to the relevant narrow range model. 

**Figure 11:**
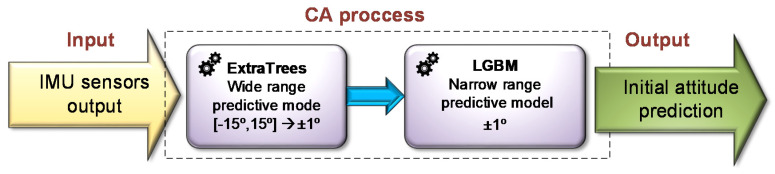
MLCA pyramidal process with the most accurate wide and narrow range regression models.

To summarize the presented experimental results, the prediction performance of the wide range MLCA models is proven to be more than sufficient to direct and focus on the relevant tested narrow range of range of 0–1° with overall accuracy of up to less than 0.2°. Thus, the overall accuracy achievable by using the proposed MLCA is determined by the performance of the ML models for the narrow range stage. The MLCA in the narrow range tests outperformed the traditional CA with an accuracy improvement for new test angle by 71% for the roll and 45% for pitch when using the LightGBM method. Given these results, it is possible to compose the best preforming ML models for each of the pyramidal methodology stages; the ExtraTrees model for the wide range predictions and the LightGBM model for the stage of narrow range predictions. This result is illustrated in Figure 11.

## 5. Conclusions

This research aimed to show the ability to implement a machine learning-based coarse alignment process, namely MLCA, instead of the traditional CA with the goal to improve the latter’s performance. To that, a MLCA methodology was proposed and derived. To evaluate the proposed MLCA methodology, the MLCA method’s effectiveness has been verified by simulation and field experiments. Running on representative data sets of stationary INS alignment scenarios, both simulated and experimental, the results of MLCA show the ability to predict the roll and pitch angles in the wide range of angles with a satisfactory accuracy to enable a narrow range evaluation. Using this two-step MLCA methodology showed better results than the traditional CA.

Classical CA keeps the same level of performance both in a narrow and wide range. For MLCA, wide range is used to direct and focus on the narrow range CA to yield the roll and pitch estimation. Therefore, high accuracy is required only in the narrow range with allowable accuracy to access it from the wide range. In the narrow range, both simulation and experimental test results showed the advantage of predicting the coarse alignment angles by the ML models over the classical method. While running in the narrow range, it was reflected in shorter prediction time to achieve the same traditional CA results and with the same CA prediction time to obtain method much better accurate solution with 71% accuracy improvement shown in field experiments.

It was shown that the overall time required in MLCA to obtain the same accuracy is much lower than the traditional CA, and its overall accuracy is better. Thus, the proposed MLCA can be applied as easily as the traditional one with better performance. The improved accuracy and required time for alignment are particularly critical for platforms operating in pure inertial navigation and when time-constraints are posed on the alignment time.

## Figures and Tables

**Table 1:** Statistical Features.

Feature	Definition	Expression
Mean	The mean value of each window	$\frac{1}{N}\sum_{i=1}^{N}x_{i}$
Standard deviation	the deviation of the mean for each window	$\sqrt{\frac{\sum_{i=1}^{N}(x_{i}-\bar{x}{)}^{2}}{N-1}}$
Variance	the variation of each window	$\frac{1}{N}\sum_{i=1}^{N}(x_{i}-\bar{x}{)}^{2}$
median	The middle value of each window	$Med(X)=\{\begin{array}{ll} X\left[\frac{N}{2}\right] & \mathrm{if}\;N\;\mathrm{is}\;\mathrm{even}\\ \frac{(X\left[\frac{N-1}{2}\right]+X\left[\frac{N+1}{2}\right]}{2} & \mathrm{if}\;N\;\mathrm{is}\;\mathrm{odd} \end{array}$
Minimum value	The minimum value in each window	$\underset{i\in N}{min\;}x_{i}$
Absolute of the minimum value	The absolute value of the minimum value in each window	$\left|\underset{i\in N}{min\;}x_{i}\right|$
Maximum value	The maximum value in each window	$\underset{i\in N}{\max\;}x_{i}$
Absolute of the maximum value	The absolute value of the maximum in each window	$\left|\underset{i\in N}{\max\;}x_{i}\right|$
Entropy	The amount of regularity and the unpredictability	$-\sum_{i=1}^{N}x_{i}{}^{2}\log(x_{i}{}^{2})$
Skewness	The asymmetry of the probability distribution	$\frac{\sum_{i=1}^{N}(x_{i}-\bar{x}{)}^{3}/N}{\sigma_{x}{}^{3}}$
Kurtosis	The “tailedness” of the probability distribution	$\frac{\sum_{i=1}^{N}(x_{i}-\bar{x}{)}^{3}/N}{\sigma_{x}{}^{4}}-3$
Energy	The sum of squares of values	$\sum_{i=1}^{N}x_{i}{}^{2}$
Amplitude	The absolute difference between the minimum and the maximum values in each window	$\left|\underset{i\in N}{\max\;}x_{i}-\underset{i\in N}{min\;}x_{i}\right|$
Histogram	The values of 4 bins histogram in each window	

**Table 2:** Time-domain features.

Feature	Definition
Number of peaks	The number of peaks with a defined minimum peak height and a minimum distance between peaks.
Zero-crossing rate (ZC)	The number of sign changes in the window

**Table 3:** Frequency-domain features.

Feature	Definition	Expression
Mean spectral energy	The mean of the signal power spectrum using a one-dimensional discrete Fourier Transform.	$\frac{1}{N}{\sum_{k=0}^{N-1}\left|x_{k}\right|}^{2}$ where: $x_{k}=\sum_{n=0}^{N-1}x_{n}e^{-\frac{2\Pi i}{N}kn}$

**Table 4:** Cross-correlation features.

Feature	Definition
Accelerometer axis correlation	The cross-correlation coefficient between the acceleration sensors binary combinations of acceleration axis *x*, *y*, and *z*

**Table 5:** CA narrow range errors after 3 s of prediction time for 0.01° train set resolution.

	MAE (mrad)		Relative Improvement	
Method	Roll	Pitch	Roll	Pitch
Classical method	0.336 (±0.458)	0.323 (±0.415)		
RF	0.254 (±0.175)	0.245 (±0.187)	39.89%	42.27%
XGBoost	0.242 (±0.184)	0.245 (±0.187)	42.75%	42.25%
LightGBM	0.244 (±0.18)	0.244 (±0.184)	42.32%	42.42%
CatBoost	0.247 (±0.188)	0.247 (±0.185)	41.60%	41.71%
ExtraTrees	0.249 (±0.178)	0.244 (±0.182)	40.99%	42.35%

**Table 6:** CA wide range MAE (mrad) after 3 s of prediction time.

	0.5° Train Set Resolution		1° Train Set Resolution	
Method	Roll MAE	Pitch MAE	Roll MAE	Pitch MAE
Classical method	0.337 (±0.461)	0.326 (±0.417)	0.337 (±0.461)	0.326 (±0.417)
RF	1.960 (±1.528)	2.249 (±1.112)	3.610 (±2.556)	3.245 (±1.690)
XGBoost	2.031 (±1.562)	2.262 (±1.109)	4.923 (±3.084)	4.360 (±2.376)
LightGBM	1.369 (±1.206)	1.707 (±1.770)	3.548 (±2.029)	4.299 (±3.017)
CatBoost	1.023 (±0.897)	1.132 (±0.961)	3.195 (±2.452)	2.626 (±1.849)
ExtraTrees	1.050 (±0.814)	0.651 (±0.467)	1.851 (±1.593)	0.952 (±0.467)

**Table 7:** CA errors in narrow range experiment for 1 s prediction time.

	MAE (mrad)		Relative Improvement	
Method	Roll	Pitch	Roll	Pitch
Classical method	0.233 (±0.154)	0.255 (±0.171)		
RF	0.164 (±0.258)	0.162 (±0.237)	29.70%	36.44%
XGBoost	0.095 (±0.231)	0.072 (±0.128)	59.17%	71.83%
LightGBM	0.033 (±0.142)	0.056 (±0.208)	85.89%	78.03%
CatBoost	0.076 (±0.198)	0.061 (±0.174)	67.27%	76.16%
ExtraTrees	0.144 (±0.247)	0.121 (±0.164)	38.22%	52.52%

**Table 8:** Narrow range experiment results for predication of a new orientation after 1 s.

	MAE (mrad)		Relative Improvement	
Method	Roll	Pitch	Roll	Pitch
Classical method	0.177 (±0.125)	0.153 (±0.105)		
RF	0.053 (±0.009)	0.084 (±0.001)	70.30%	45.07%
LightGBM	0.052 (±0.007)	0.096 (±0.013)	70.94%	36.84%
ExtraTrees	0.174 (±0.036)	0.127 (±0.063)	2.14%	16.97%

**Table 9:** Wide range experiment with new angle after 1 s.

	MAE (mrad)	
Method	Roll	Pitch
Classical method	0.597 (±0.406)	0.782 (±0.478)
RF	3.283 (±0.000)	3.431 (±2.237)
XGBoost	3.072 (±0.139)	5.012 (±0.000)
LightGBM	3.935 (±2.735)	0.997 (±0.450)
CatBoost	9.41 (±4.526)	5.202 (±0.931)
ExtraTrees	2.076 (±0.215)	1.092 (±0.519)
